# Metabolic and morphometric changes in Indonesian cynomolgus monkeys (Macaca fascicularis) fed an atherogenic diet composed of locally sourced ingredients

**DOI:** 10.14202/vetworld.2018.1609-1617

**Published:** 2018-11-22

**Authors:** Sri Rahmatul Laila, Dewi Apri Astuti, Irma Herawati Suparto, Ekowati Handharyani, Dondin Sajuthi

**Affiliations:** 1 Department of Anatomy, Physiology and Pharmacology, Faculty of Veterinary Medicine, Bogor Agricultural University, Bogor, Indonesia; 2 Primate Research Center, Bogor Agricultural University, Bogor, Indonesia; 3 Department of Nutrition and Feed Technology, Faculty of Animal Science, Bogor Agricultural University, Bogor, Indonesia; 4 Department of Chemistry, Faculty of Mathematics and Natural Sciences, Bogor Agricultural University, Bogor, Indonesia; 5 Department of Veterinary Clinic, Reproduction and Pathology, Faculty of Veterinary Medicine, Bogor Agricultural University, Bogor, Indonesia

**Keywords:** atherogenic diet, carotid artery, cynomolgus, ultrasonography

## Abstract

**Background and Aim:**

This study was designed to determine the effects of a new atherogenic diet formulated at Institut Pertanian Bogor (IPB) (Bogor, Indonesia) on metabolic, morphometric, and carotid artery imaging of cynomolgus monkeys.

**Materials and Methods:**

A total of 20 adult male cynomolgus monkeys fed IPB-1 atherogenic diet for 1 year. Total plasma cholesterol (TPC), high-density lipoprotein (HDL) cholesterol, low-density lipoprotein (LDL) cholesterol, triglycerides, and morphometric measurements were evaluated at baseline and monthly during the study. Carotid plaques and intima-media thickness (IMT) were measured using ultrasonography at baseline and after 8 months of treatment.

**Results:**

This diet increased TPC, LDL, and TPC/HDL ratio and induced carotid atherosclerosis in this model. The TPC, LDL, and TPC/HDL ratio were positively associated; however, HDL was negatively associated with carotid plaques and IMT.

**Conclusion:**

The IPB-1 atherogenic diet formulated with locally and readily available ingredients provides an economically and scientifically feasible monkey model to study atherosclerosis in Indonesia and Southeast Asia.

## Introduction

Atherosclerosis is the major cause of morbidity and mortality in cardiovascular disease (CVD) [1]. In Indonesia, CVD has been reported as the leading cause of death during 2000-2012 [2]. CVD led to an estimated economic loss of approximately 1.77 trillion US$ in 2010 [3], and this number will increase significantly in 2025. Since lots of herbal components may influence atherosclerosis, many Indonesian researchers have become interested in determining the effects of those compounds. Those researches required an animal model. The Indonesian cynomolgus monkey (*Macaca fascicularis*) is an appropriate animal model susceptible to diet-induced atherosclerosis [4,5].

The purpose of the present study was to develop and test an atherogenic diet generated from locally produced ingredients to mimic those in previous studies of cynomolgus monkeys. Initially, we imported the ingredients for a western atherogenic diet from the Primate Research Center (PRC) at the Wake Forest School of Medicine. But then, we developed several atherogenic diets using locally sourced ingredients at the PRC of Institut Pertanian Bogor (PRC-IPB), Indonesia. The IPB-1 atherogenic diet was the proper diet formulated from rice bran, beef tallow, soya meal, sugar, fresh egg yolk, fish meal, coconut oil, corn oil, corn meal, a trace of minerals, and vitamins. The diet was formulated with ingredients to be similar to those eaten by Southeast Asian people, especially Indonesian people. The ingredients of this diet are widely available and expected to be stable over time. This diet has a high cholesterol content and resulted in high total plasma cholesterol (TPC) levels of ~400 mg/dl and balanced nutrition during 1-year feeding [6].

In the study reported here, individual monkeys had variable responses to the dietary cholesterol in IPB-1 atherogenic diet, consistent with previous studies using western atherogenic diets in the cynomolgus monkey [7,8], and known variable responses in humans [9-11]. TPC and other plasma lipid measures are known predictors of risk for the development of atherosclerosis. Carotid ultrasonography (USG) is clinically useful in identifying atherosclerotic changes in the arterial wall in human. The presence of plaque can be determined by measuring the shape, echogenicity changes, and intima-media thickness (IMT) of the arterial wall [12,13].

This study was designed to evaluate the capability of IPB-1 atherogenic diet to induce atherosclerotic responses detectable by metabolic change and carotid USG, and also to explore the relationships between those phenotypes in cynomolgus monkeys as an atherosclerotic animal model.

## Materials and Methods

### Ethical approval

All works were approved by the Animal Care and Use Committee from PRC-IPB (ACUC IPB PRC-14-B003). The PRC-IPB is accredited by the Association for Assessment and Accreditation of Laboratory Animal Care International (AAALAC International).

### Animals

The subjects of this study were 20 male adult cynomolgus monkeys that were obtained and maintained at the animal facility at PRC-IPB, Indonesia. They ranged in age from 7 to 9 years (estimated by dental examination) with average body weight (BW) of 4-7 kg. Animals were housed in individual cages positioned proportionally, so they could see and hear each other.

### Study design

Before this study, monkeys had consumed IPB-1 atherogenic diet (containing 0.28-0.29 mg/Cal cholesterol) for 1 year and produced hypercholesterolemia [6]. In this study (2^nd^ year), animals continued to consume IPB-1 atherogenic diet (containing 0.28 mg/Cal cholesterol daily) (Table-1) to determine the development of atherosclerotic lesions. Baseline characteristics of BW, waist circumference (WC), trunk length (TL), TPC, high-density lipoprotein (HDL) cholesterol, triglycerides (TGs), blood glucose (Glu), and the presence of plaque in the carotid artery by USG were determined in all monkeys before treatment. All the measurements were done while the animals were sedated with ketamine HCl (Ket-A-100^®^, Senasa, Peru) (10-12 mg/kg BW).

**Table-1:** Ingredients and nutrient composition of IPB-1 atherogenic diet.

Ingredient	% dry matter	Nutrient composition (%)
Wheat flour	42	Dry matter (86.72)
Sugar	9	Protein (14.96)
Egg yolk	10	Lipids (19.82)
Beef tallow	5	Carbohydrate (49.46)
Coconut oil	8	Fiber (2.48)
Corn oil	2	
Corn meal	8	
Rice bran	3	
Soya meal	6	
Fish meal	5	
Mineral mix	1	
Cellulose	1	
Vitamin mix	1	

The monkeys consumed the IPB-1 atherogenic diet for 1 year more. The diet was prepared at PRC-IPB weekly and stored in a freezer. The monkeys received 180-200 g of diet that contained 120 Cal/kg BW, with total cholesterol 113.8 mg or 0.28 mg cholesterol/Cal daily comparable to western diet to induced atherosclerosis in cynomolgus monkeys [7]. All monkeys were fed twice a day (at 08.00 and 15.00), one piece of banana (70 g) with water provided *ad libitum*.

### Morphometric measurement

Morphometric measurements were recorded from all monkeys monthly. BW (kg) was measured using digital scales. WC (m) was measured by flexible tape that placed around the monkey’s abdomen at the level of the umbilicus. TL (m) was measured by a caliper that placed from the suprasternal notch onto the symphysis pubis. All measurements were made twice [14]. Adiposity index was calculated as the BW (kg) divided by TL squared (m^2^). Veterinarians performed physical examinations and reported any health problem that occurred in monkeys during the treatment.

### Metabolic measurements

Blood samples for the determination of lipid profiles and Glu were taken monthly from all animals. Blood samples were collected from their femoral vein into ethylenediaminetetraacetic acid Vacutainer. Whole blood was centrifuged, and lipid profiles (HDL, TGs, and TPC) and Glu were determined using Photometer 5010 (RIELE™, Berlin, Germany) and presented in mg/dl. The low-density lipoprotein (LDL) cholesterol was calculated using the Iranian formula (TPC/1,19 + TG/1,9 - HDL/1,1 - 38) [15], and a ratio of TPC/HDL was also determined. In the adaptation period, the monkeys showed different responses to dietary cholesterol. They were divided into three categories based on TPC levels: Hyporesponsive (TPC <200 mg/dl), intermediate responsive (TPC 200-400 mg/dl), and hyperresponsive (TPC >400 mg/dl) group for additional analyses.

### Carotid USG

Both the lateral sides of the monkeys’ neck were shaved to allow scanning the left and right carotid arteries. The USG was performed by a trained sonographer using B-mode USG (Philips Envisor, Eindhoven, the Netherlands). An 8 MHz frequency USG transducer with the linear probe was used to measure the plaque. Location of the bifurcation (BIF) of the carotid artery was determined and scanned, then, the common carotid artery (CCA) was also scanned from the proximal to distal [12,13]. In longitudinal view, the adventitia layer appears as bright, media layer as dark gray and lumen as black. The BIF and CCA were measured, and images were captured for further interpretation. Atherosclerotic lesions were analyzed through the determination of echogenicity (opacity), homogeneity, continuity, and IMT of artery wall [13]. The plaque was defined as areas where intimal-media appeared bright (an increase of opacity), inhomogeneous, and form a focal thickening in artery wall with minimal thickness 0.3 mm.

### Statistical analysis

The Kolmogorov–Smirnov test was used to determine the normality of the data. Data are reported as mean±standard deviation. Plasma lipid values obtained over time were used to calculate the mean value of the measurements. The differences between baseline and treatment were analyzed by paired t-test. Multivariate analysis (ANOVA) was performed to determine the influence of responsiveness (hypo-, intermediate-, and hyper-responsive group) for all variables. Association between carotid IMT and presence of plaque with blood lipid profiles and other variables was evaluated by Pearson or Spearman correlation as appropriate.

## Results

### Morphometric and metabolic profiles

In Table-2, we have summarized the results of the clinicopathologic observations taken both before the initiation of the treatment (baseline) and the end of the treatment. The mean of TPC, HDL, TG, and Glu at the baseline was 234, 45, 35, and 44 mg/dl, respectively. Prevalence of the CCA plaque was 25%, and BIF plaque was 45%. In general, the animals were in good health. Table-2 shows that IPB-1 atherogenic diet raised the TPC, TGs, and also Glu and decreased the HDL. Other than that, IPB-1 atherogenic diet increased plaques in carotid arteries of these monkeys. The monkeys in this study lost weight and adiposity index during the treatment compared with baseline but still within the normal range. The monkeys showed a different response to dietary cholesterol, so for future analyses, they were divided into three groups: Hypo-, intermediate-, and hyper-responsive animals with the total of animals were four, eight, and eight animals, respectively.

**Table-2:** Morphometric and metabolic profiles of 20 monkeys at the baseline and the end of treatment.

Variables	Baseline (Mean±SD)	End of the Treatment (Mean±SD)	p-value
BW (kg)	5.68±0.67	4.81±0.78	0.000
AI	64.40±8	60.70±7	0.013
TPC (mg/dl)	234±99	323±148	0.031
HDL (mg/dl)	45±16	39±12	0.034
TPC/HDL	6.4±4	9.3±5	0.010
TGs (mg/dl)	35±17	51±18	0.003
Glu (mg/dl)	44±8	55±10	0.001
Animals with CCA plaque (%) n=20	25	50	
Animals with BIF plaque (%) n=20	45	75	

BW=Body weight, AI=Adiposity index, TPC=Total plasma cholesterol, HDL=High-density lipoprotein, LDL=Low-density lipoprotein, TGs=Triglycerides, Glu=Blood glucose, CCA=Common carotid artery, BIF=Bifurcation carotid

**Figure-1:**
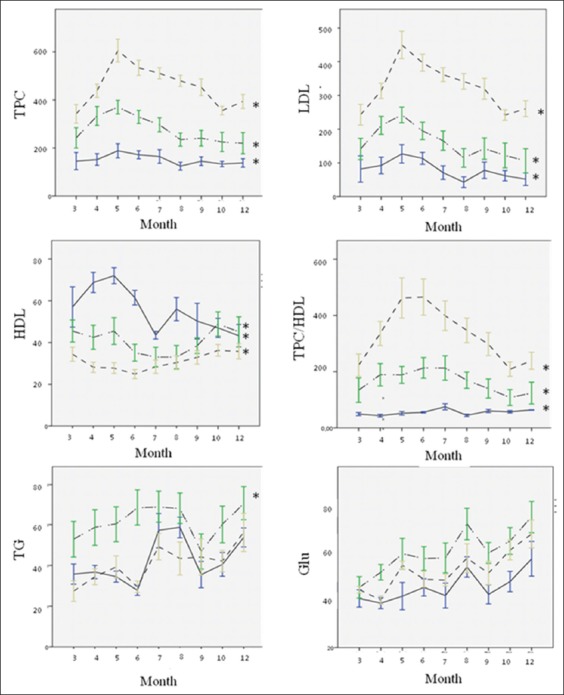
Mean of metabolic profiles among three different responsiveness plotted over time of the treatment period. TPC=Total plasma cholesterol; LDL=Low-density lipoprotein; HDL=High-density lipoprotein; TGs=Triglycerides; Glu=Blood glucose. (‒): hyporesponsive, (-‒--‒-): intermediate responsive, (- - -): hyperresponsive. *significant at p<0.05.

We have summarized BW, adiposity index (AI), plasma lipid profiles, and Glu of the three groups of the monkeys during 9 months treatment in Table-3. There was no difference between three groups respond for BW and AI. Still, Glu in the intermediate responsive group was significantly higher compared with hypo- and/or hyper-responsive group (p<0.05). Table-3 also shows that hyper-responsive group has the highest value of LDL as well as TPC/HDL, followed by intermediate- and hypo-responsive group (p<0.05). As expected, HDL was highest in hypo-responsive group and lowest in hyper-responsive group. Interestingly, TGs in the intermediate responsive group were significantly higher compared with hypo- and/or hyper-responsive group (p<0.05). Mean of metabolic profiles for three groups is shown in Figure-1.

**Table-3:** Morphometric and metabolic profiles of the three groups of the monkeys during 9 months’ treatment.

Variable	Groups		

	Hyporesponsive	Intermediate responsive	Hyperresponsive
BW (kg)	4.92±0.60	5.35±0.75	5.10±0.47
AI	57.20±8	58.50±9	58.70±4
Glu (mg/dl)	45±4.40^3^	61±12.10^1^	54±7.40^2^
TPC (mg/dl)	155±34.70^3^	286±68.70^2^	471±57.30^1^
HDL (mg/dl)	57±5.10^1^	41±12.90^2^	30±6.30^3^
LDL (mg/dl)	80±50^3^	160±92^2^	325±106^1^
TPC/HDL	2.78±0.69^3^	8.22±5.10^2^	16.50±7.80^1^
TGs (mg/dl)	43±5.80^2^	65±18.80^1^	40±12.30^2^

BW=Body weight, AI=Adiposity index, TPC=Total plasma cholesterol, HDL=High-density lipoprotein, LDL=Low-density lipoprotein, TGs=Triglycerides, Glu=Blood glucose. Different superscript significant at p*<*0.05

Figure-1 shows that, at the 6^th^ month of feeding, TPC was increased in all groups and then decreased over time, contrary with HDL. The value of TPC at the 9^th^ month was quite similar to it at the 3^rd^ month and was maintained at the same level for the next 3 months until the end of treatment. Hyper- and intermediate-responsive animals achieved TPC values above 200 mg/dl after 3 months of treatment. TPC in hyper-responsive animals remained above 400 mg/dl, whereas in hypo-responsive animals, TPC value does not exceed 200 mg/dl until the end of treatment (p<0.05). The trend of LDL and TPC/HDL was presented to be analogous with TPC.

### Carotid plaques and IMT by USG

**Figure-2:**
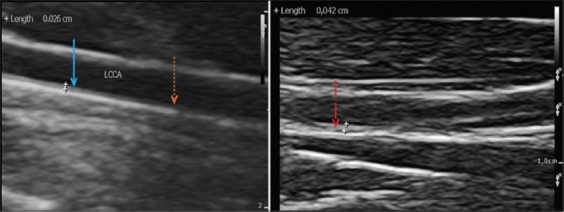
Common carotid artery ultrasound images of treated cynomolgus monkeys. A=hyporesponsive group, B=hyperresponsive group. (->): intimal xanthoma, (->): pathological intimal thickness, (---->): normal artery.

We reported here that this diet increases the presence of the plaque both in the CCA and BIF of carotid arteries. In these results, the carotid IMT of the monkeys was 0.21-0.71 mm, with the minimum IMT of 0.3 mm indicated to atherosclerosis plaques. The IMT of 3 group monkeys is shown in Table-4. Formation of thickened atherosclerosis lesions/pathologic intimal thickness by USG in the left CCA was higher than in the right CCA (45% and 25%, respectively). Carotid BIF showed evidence of severe plaques. At the left BIF, the presence of plaque was 70% and at the right BIF was 60%. Table-4 shows that 25% of monkeys in the hypo-responsive group had CCA plaques; moreover, 50% of monkeys in the intermediate responsive group and 75% of monkeys in the hyper-responsive group had plaques at CCA. Ultrasound images of atherosclerosis lesion on CCA are shown in Figure-2. Similar findings were observed for the BIF, the highest form of plaques was in the hyper-responsive group, and the smallest was in the hypo-responsive group.

**Table-4:** Intimal media thickness of carotid arteries of monkeys fed with IPB-1 atherogenic diet.

Variables	Groups		

	Hyporesponsive	Intermediate responsive	Hyper responsive
CCA IMT R (mm)	0.248±0.01^2^	0.299±0.02^1^	0.326±0.03^1^
CCA IMT L (mm)	0.275±0.03	0.325±0.06	0.330±0.07
BIF IMT R (mm)	0.283±0.02^2^	0.364±0.06^12^	0.404±0.09^1^
BIF IMT L (mm)	0.318±0.06	0.366±0.05	0.408±0.09
Animals with CCA plaque (%)	25% (n=4)	50% (n=8)	75% (n=8)
Animals with BIF plaque (%)	50% (n=4)	75% (n=8)	87.5% (n=8)

CCA=Common carotid artery, BIF=Bifurcation carotid, IMT=Intima-media thickness, R=right, L=Left. Superscript^1, 2, 3^ in line significantly different (p<0.05)

### Correlation between morphometric, metabolic, and carotid USG measures

The correlation between morphometric and plasma lipid profiles with plaques and IMT of the carotid artery is summarized in Tables-5 and 6. Table-5 shows TPC, TPC/HDL, and LDL significantly associated with the presence of plaques in carotid arteries. Increasing of that three variables would increase the presence of plaque in both the right and left carotid arteries. There was no association between AI, TG, and Glu with the presence of plaques detected by USG.

**Table-5:** The correlation between morphometric and plasma lipid profiles with the presence of plaques in the carotid artery of monkey fed with IPB-1 atherogenic diet.

Variable	Spearman correlation (n=20)			

	Right CCA plaque	Left CCA plaque	Right BIF plaque	Left BIF plaque
BW	r=0.341	r=0.471*	r=−0.027	r=0.274
	p=0.142	p=0.036	p=0.911	p=0.242
IA	r=0.265	r=0.218	r=−0.142	r=0.038
	p=0.259	p=0.356	p=0.552	p=0.874
TPC	r=0.303	r=0.497*	r=0.584**	r=0.454*
	p=0.194	p=0.026	p=0.007	p=0.044
HDL	r=−0.351	r=−0.218	r=−0.195	r=−0.284
	p=0.130	p=0.355	p=0.410	p=0.225
LDL	r=0.303	r=0.497*	r=0.584**	r=0.435
	p=0.194	p=0.026	p=0.007	p=0.055
TPC/HDL	r=0.416	r=0.532*	r=0.531*	r=0.473*
	p=0.068	p=0.016	p=0.016	p=0.035
TGs	r=−0.284	r=0.209	r=0.053	r=0.180
	p=0.225	p=0.376	p=0.824	p=0.448
Glu	r=0.209	r=0.245	r=0.267	r=0.361
	p=0.377	p=0.298	p=0.256	p=0.118

BW=Body weight, AI=Adiposity index, TPC=Total plasma cholesterol, HDL=High-density lipoprotein, LDL=Low-density lipoprotein, TGs=Triglycerides, Glu=Blood glucose.

* p<0.05,

** p<0.01. CCA=Common carotid artery, BIF=Bifurcation carotid

In Table-6, TPC, TPC/HDL, and LDL were also positively associated with the IMT of carotid arteries. It means that IMT will increase if those variable increases. Other than that, HDL was negatively associated with IMT of the carotid artery. Decreasing of HDL will increase IMT of carotid arteries in monkeys.

**Table-6:** The correlation between morphometric and plasma lipid profiles with IMT carotid artery of monkey fed with IPB-1 atherogenic diet.

Variable	Pearson Correlation (n-20)			

	Right CCA IMT	Left CCA IMT	Right BIF IMT	Left BIF IMT
BW	r=0.263	r=0.535*	r=0.299	r=0.448*
	p=0.262	p=0.015	p=0.201	p=0.047
IA	r=0.150	r=0.355	r=0.161	r=0.276
	p=0.529	p=0.125	p=0.497	p=0.238
TPC	r=0.295	r=0.539*	r=0.534*	r=0.552*
	p=0.207	p=0.014	p=0.015	p=0.012
HDL	r=−0.602**	r=−0.321	r=−0.444*	r=−0.221
	p=0.005	p=0.167	p=0.050	p=0.350
LDL	r=0.254	r=0.514*	r=0.523*	r=0.564**
	p=0.280	p=0.021	p=0.018	p=0.010
TPC/HDL	r=0.451*	r=0.548*	r=0.555*	r=0.438
	p=0.046	p=0.012	p=0.011	p=0.053
TGs	r=0.127	r=0.235	r=0.095	r=0.047
	p=0.593	p=0.318	p=0.890	p=0.844
Glu	r=0.282	r=0.307	r=0.402	r=0.105
	p=0.228	p=0.188	p=0.079	p=0.059

BW=Body weight, AI=Adiposity index, TPC=Total plasma cholesterol, HDL=High-density lipoprotein, LDL=Low-density lipoprotein, TGs=Triglycerides, Glu=Blood glucose.

* p<0.05,

** p<0.01. CCA=Common carotid artery, BIF=Bifurcation carotid

## Discussion

### Diet, morphometric, and metabolic profiles

The IPB-1 atherogenic diet was formulated from wheat flour, sugar, beef tallow, egg yolk, coconut oil, corn oil, corn meal, rice bran, soy meal, fish meal, trace minerals, cellulose, and vitamin mix. The ingredient was high in cholesterol, carbohydrate, and fat [16]. Diets on high cholesterol and saturated fatty acid are associated with elevated serum cholesterol concentration. Egg yolk is a major source of cholesterol. Consuming egg yolk will increase 10% of LDL in plasma [17]. Coconut oil has a very high saturated fatty acid (86.50%) and low polyunsaturated fatty acids [18]. Corn oil has high polyunsaturated fatty acid that added to balance the fatty acid supply. Saturated fatty acids increase plasma cholesterol, and polyunsaturated fatty acids will lower plasma cholesterol, whereas monounsaturated fatty acids do not affect plasma cholesterol [19]. This diet also has high energy (120 Cal/kg BW). A high energy intake contributed to induce the risk factor of CVD in human and animal model [20]. We could see that carbohydrate was the main energy source in this diet. High level of carbohydrate intake decreases HDL and increases the TGs [21].

As we predicted, the IPB-1 atherogenic diet increased the TPC of animal model *M. fascicularis* in this study. The mean of TPC in 9 months’ treatment was 323 mg/dl. Ordinarily, the normal value of TPC in *M. fascicularis* was 176 mg/dl [22]. We recognized three different responses that called hypo-, intermediate-, and hyper-responsive to dietary cholesterol in this study. Hyporesponsiveness and hyperresponsiveness have been reported in some studies of several monkey species. It occurred among the monkeys that fed with similar cholesterol-containing diet. Clarkson *et al*. [7] reported the hypo- and hyper-responsive in cynomolgus monkeys that fed with the western diet containing 150 mg or 0.35 mg/cal cholesterol. This western diet has been used in lots of studies both in male or female cynomolgus monkeys [8,23]. In human, variance in responsiveness was explained by serum total of cholesterol and HDL levels [24]. In addition, Glatz *et al*. [10] also reported that hyporesponders in man were determined by low TPC, high HDL, and low body mass index, the opposite with hyperresponders.

Variable of TPC in this study can exist because of the considerable variability in dietary cholesterol absorption. As described previously, this diet had a good digestibility value [6]. Responsiveness to dietary cholesterol was also influenced by the genetic factor [8]. Genetic diversity in the 3’UTR of LDLR gene was studied using isolated DNAs from cynomolgus monkeys, which have different responses to this atherogenic diet [25]. Other than that, Glatz *et al*. [10] reported that different responses in the value of TPC were caused by the different capacities of LDL synthesis and LDL catabolic rate in the liver. Difference responsiveness was also inherited by offspring.

TPC in hypo-responsive monkeys that consumed IPB-1 atherogenic diet remained <200 mg/dl until the end of treatment. At the same time, TPC in hyper-responsive monkeys was steady above 400 mg/dl. It almost resembles to Clarkson *et al*. [7] study using a western diet that hypo- and hyper-responsive can be determined after 2 months feeding with mean TPC in hypo-responsive monkeys which was little higher (221 mg/dl) and mean TPC in hyper-responsive monkeys which was around 423 mg/dl throughout 2 years feeding. In our recent data with IPB-1 atherogenic diet, LDL and ratio TPC/HDL graph looks similar to TPC and contrary with HDL as well as in other studies. LDL is the primary atherosclerotic lipoprotein, whereas HDL is anti-atherosclerotic lipoprotein. LDL should remain the primary therapeutic targets for patients at risk for CVD [26]. Both HDL and LDL can be used to predict the risk factors of atherosclerosis through a mathematical model [27]. Nevertheless, there is insufficient evidence to support the use of therapeutic interventions to raise low serum levels of HDL [28].

Intermediate response group was high in Glu and TGs. TGs are neutral fat, which is the main component of fat in animal cells. High level of Glu could induce a high level of TGs and, in conclusion, increase the LDL in Indonesian young people [21]. In this study, there was no significant difference between BW and AI between all the groups. A study with western diet also reported that there was no effect of the diet on the adiposity index in both male and female cynomolgus monkeys [7,29]. Furthermore, we recorded that alopecia, gingivitis, and dental caries were developed in a few monkeys, even though they did not show any major complication. Long-term singly housed contributes to increasing hair cortisol and induced alopecia in macaques [30].

We sum up that the IPB-1 atherogenic diet could induce similar characteristic in body measurement and plasma lipid profiles as well as by western atherogenic diet and suggested that responsiveness could be a good sign to choose the individual monkeys as an atherosclerotic model in both for western and IPB-1 atherogenic diet. If there was no increase of TPC in the first 3 months of feeding, the monkeys might not produce hypercholesterolemia, although for long-term feeding.

### Carotid USG

Imaging of the arteries will become increasingly useful, justified, and available to determine atherosclerosis condition [12]. The goal of imaging is similar to that for examination of the plaque by pathological techniques. In clinical trials, the measurement of atherosclerosis most frequently defined in carotid artery [31]. Our present study proved that 1-year feeding with IPB-1 diet increases the presence of plaque (by USG), in which the prevalence of plaques in the left arteries was higher than the right arteries in both common carotid and carotid BIF. These results differ from those reported in rhesus macaques that high-fat diet-induced plaques are more worse in the right carotid arteries [32].

The cholesterol responsiveness showed to be associated with the grade of plaque that formed on the arterial wall. Arteries of the hypo-responsive group have the mildest lesions, followed by intermediate responsive, and the most severe lesions are in a hyper-responsive group. As reported by Zeng *et al*. [32], lowering the TPC in rhesus macaques will inhibit increasing of the IMT. On the other hand, remaining high TPC will increase the IMT. These results could approach like what happened on the histopathological finding in hypo- and hyper-responsive monkeys that consumed the western diet [7].

The IMT of carotid was usually measured at the CCA because of its easier accessibility, whereas carotid atherosclerosis predominantly occurs earliest downstream in the BIF (and often only in the BIF) [33]. The standard value of common carotid IMT that indicates the presence of atherosclerotic plaque in animal model *M. fascicularis* has not been mentioned before. We determined that cynomolgus carotid plaques in this study were presented at the IMT at least 0.3 mm. In *Macaca mulatta*, IMT of normal BIF carotid was 0.3-0.4 mm [32]. In human, normal common carotid IMT has been established approximately 0.5-1.1 mm; thus, values 1.1 mm are considered to an atherosclerotic plaque, and its correlates linearly with the number of atherosclerotic risk factors [34].

Our data showed that TPC, TPC/HDL, and LDL had a significant correlation with the presence of plaques and IMT of CCA. This finding reflects the ability of lipid profiles to predict carotid IMT. This results almost similar to Yang *et al*. [35] and Tamada *et al*. j[36] that reported the ratio of serum LDL and HDL associated with increased of carotid IMT and may be useful for predicting the presence of carotid plaque in a Chinese population. The presence of carotid plaque is stronger than IMT as a predictor of coronary artery disease [37]. Actually, increasing of arterial IMT may be due to adaptation to blood flow, wall tension, or lumen diameter. It caused non-atherosclerotic processes such as smooth muscle cell hyperplasia, fibrocellular hypertrophy, or compensatory of arterial remodeling [38]. In addition, IMT also affected by blood pressure, especially in the left carotid [39].

## Conclusion

The IPB-1 atherogenic diet had induced the similar characteristics of atherosclerosis disease regarding lipid profiles and increased the presence of plaque in carotid arteries (using USG) as well as another atherogenic diet in monkey studies and also mimics those in human. Cholesterol responsiveness in this study influences the level of LDL, HDL, TG, and Glu, likewise the presence of the plaque by USG. The presence of plaques and IMT was positively associated with plasma TPC, LDL, and TPC/HDL ratio and was negatively associated with HDL. Consequently, from all the findings of this study, IPB-1 atherogenic diet can be used to study atherosclerosis in cynomolgus monkey model.

## Authors’ Contributions

DS and DAA designed this study. SRL conducted the experimental and wrote the manuscript. DAA made and analyzed the IPB-1 diet composition. IHS, SRL, and EH interpreted the blood profiles and ultrasound images. All authors read and approved the final manuscript.
